# Validation of the Korean Version of the Cancer Fatigue Scale in Patients with Cancer

**DOI:** 10.3390/healthcare11121796

**Published:** 2023-06-19

**Authors:** Haneul Lee, Eun Young Park, Ji Hyun Sung

**Affiliations:** 1 Department of Physical Therapy, College of Health Science, Gachon University, Incheon 21936, Republic of Korea; leehaneul84@gachon.ac.kr; 2 College of Nursing, Gachon University, Incheon 21936, Republic of Korea; parkeunyoung@gachon.ac.kr; 3 College of Nursing, Kosin University, 262 Gamcheon-ro, Seo-gu, Busan 49267, Republic of Korea

**Keywords:** cancer fatigue scale, cancer-related fatigue, validation study, Korean version

## Abstract

Cancer-related fatigue is a highly prevalent and distressing symptom that negatively affects the quality of life of patients in all stages of cancer, including survivors. The Cancer Fatigue Scale (CFS) is a 15-item multidimensional instrument with the potential to enhance comprehension of fatigue. This study aimed to translate the original English version of the CFS into Korean and establish the validity and reliability of the translated version. A cross-sectional descriptive design was used to translate and validate the CFS in Korean. Factor analyses were performed to understand and establish construct and convergent validity with the Brief Fatigue Inventory (BFI), Functional Assessment of Chronic Illness Therapy-Fatigue (FACT-F), and European Organization for Research and Treatment of Cancer Quality of Life Questionnaire Core-30 (EORTC QLQ-C30). The CFS demonstrated good internal consistency (Cronbach’s alpha coefficient for all 15 items = 0.806); the Kaiser–Meyer–Olkin Measure of Sampling Adequacy was found to be 0.897, and Bartlett’s test of sphericity was significant (*p* < 0.001). Moderate correlations were found between BFI, FACT-F, and EROTC QLQ-C30, indicating moderate validity. However, there were differences in factorial validity between the original scale and the Korean version, demonstrating a need for further testing in a homogenous population of cancer patients. The findings of this validation and reliability study showed that the Korean version of the CFS is a concise, reliable, feasible, and practical tool for evaluating the multidimensional aspects of cancer-related fatigue in patients with cancer.

## 1. Introduction

Cancer-related fatigue (CRF) is a prevalent and distressing symptom experienced by cancer patients during all stages of their illness and cancer survivors [1,2]. The prevalence of fatigue ranges from 25% to 99%, depending on the measurement method, treatment type, and patient population [2,3]. CRF is defined as a condition characterized by more severe, persistent, and debilitating fatigue than normal with physical and psychological manifestations, such as weakness, decreased concentration, diminished motivation, and emotional instability [4,5,6,7]. Despite its negative impact on quality of life (QoL), there is currently an absence of a gold standard questionnaire to measure CRF, and it is not commonly discussed with cancer patients [8,9]. The absence of adequate instruments has led to imprecise criteria for significant fatigue, as well as an inconsistent use of instruments [2], despite the increasing need to assess fatigue as a routine part of healthcare research and clinical practice. 

The properties of fatigue measurement scales should align with the definition of CRF. The National Comprehensive Cancer Network proposes one of the most commonly cited definitions, stating that fatigue is a distressing, persistent subjective sense of physical, emotional, and/or cognitive tiredness or exhaustion related to cancer or cancer treatment [10]. Consequently, several instruments have been developed to measure multidimensional CRF, differing in psychometric properties, administration methods, item numbers, and dimensions. Additionally, many instruments have been validated in specific cancer types or during particular treatments, limiting their generalizability across all patients with cancer. 

Previous systematic reviews [2,11] have identified two unidimensional tools, namely the Functional Assessment of Cancer Therapy-Fatigue (FACT-F) and Brief Fatigue Inventory (BFI), as meeting the most quality assessment criteria and having nearly ideal psychometric properties within a cancer population. These instruments have been translated, and their Korean versions have been validated. However, unidimensional instruments have limitations since they usually exclusively measure severity, whereas researchers and clinicians should consider other aspects, such as emotional or cognitive fatigue, when choosing a CRF scale. 

The Cancer Fatigue Scale (CFS) is a 15-item, 5-point Likert scale comprising three domains: physical, affective, and cognitive [12]. The German [13] and Chinese versions [14] of this scale have been validated in a heterogeneous cancer population, encompassing various cancer stages, treatment modalities, and Karnofsky performance scores. Moreover, the scale has been translated into Greek [15], Turkish [16], and Brazilian [17], demonstrating good reliability. Based on the review findings [11], the CFS fulfills the quality assessment criteria for content, construct, and criterion validity as well as internal consistency. However, the CFS has not yet been validated in the Korean population. Therefore, the purpose of this study was to examine the validity and reliability of the Korean version of the CFS in patients with various types of cancer.

## 2. Methods

### 2.1. Ethics Statement

The study was approved by the Gachon University Institutional Review Board (1044396-202201-HR-016-02) and four medical centers where the data were collected. This study was conducted in accordance with the Declaration of Helsinki. The participants were informed about the purpose of the study, and those who agreed to participate signed an informed consent form before the survey began. Participants were guaranteed the right to voluntarily participate in the study and their privacy.

### 2.2. Participants

A total of 209 patients with different types of cancers were recruited using a convenience sampling method from four different medical centers in Korea. All patients diagnosed with cancer were older than 18 years and could read and understand Korean. The general characteristics of the participants are described in Table 1. The mean age of the participants was 61.24 years (range, 30–98 years). The participants consisted of 98 women (46.9%) and 111 men (53.1%). The most frequent cancer site was the stomach (31.1%), which was followed by the breast (19.1%).

The sample size estimation was based on a previous study by MacCallum et al. [18] that reported that 4–10 participants per variable, with a minimum of 100 participants, are needed to ensure the stability of a factor analysis test. Therefore, a minimum of 150 patients were needed, and considering dropout, 220 questionnaires were distributed to patients with cancer. 

### 2.3. Data Collection

Data were collected between April and June 2022. The questionnaire was distributed to patients with cancer identified as eligible by oncology nurses who provided initial permission. Data collection was conducted using structured self-administered questionnaires for patients with cancer. The mean time to complete the questionnaires was approximately 15 min. A total of 220 questionnaires were distributed to four different medical centers, of which 210 were returned, resulting in a response rate of 95.5%. However, one questionnaire was incomplete; thus, 209 questionnaires were included for analysis. 

The patients self-reported their general characteristics, including age and sex, and medical information, such as the type of cancer, stage, and types of treatment they were currently receiving. 

### 2.4. Measures

#### 2.4.1. Cancer Fatigue Scale Questionnaire

The CFS is composed of three domains: physical (7 items), affective (4 items), and cognitive domain (4 items) and was originally developed by Okuyama et al. [12] to assess the nature of fatigue across these subscales experienced by patients with cancer. Each item on the scale is rated using a 5-point Likert scale, ranging from 1 (not at all) to 5 (very much). It is important to note that the scoring for the affective subscales (items 5, 8, 11, and 14) is inverted. The CFS has different maximum scores for its subscales: 28 for the physical subscale, 16 for the affective subscale, and 16 for the cognitive subscale. The total scores on the scale can range from 0 to 60, with higher scores indicating more severe fatigue [12]. The original version of the scale was reliable with Cronbach’s alpha values for physical, affective, cognitive, and total scale of 0.89, 0.79, 0.79, and 0.88, respectively [12]. The CFS is also considered to be simple and easy to complete and applicable to patients. The validity and reliability of the scale has been established in different languages [13,14,15,16,17,19].

#### 2.4.2. Korean Version of the Brief Fatigue Inventory

The Korean version of the Brief Fatigue Inventory (BFI-K) was used to assess the severity and impact of cancer-related fatigue [20]. This instrument was originally developed by Mendoza et al. to assess the severity of fatigue related to cancer or cancer treatment [21]. There are 9 items consisting of the fatigue level and how much fatigue has interfered with the patients’ life during the past 24 h. All items were scored from 0, meaning ‘not interfering’ to 10, meaning ‘completely interfering’ with activity or work. The validity and reliability of the original instrument have been established [21] and the internal consistency reliability of BFI-K was 0.96 in patients with cancer [9]. 

#### 2.4.3. Functional Assessment of Chronic Illness Therapy—Fatigue

The FACT-F is an instrument used to assess fatigue and its impact on daily activities, such as eating, sleeping, and working, in patients with cancer over the past week. The FACT-F was originally developed to meet the growing demand for a more precise evaluation of cancer-related fatigue. The FACT-F is the 13-item fatigue subscale of the Functional Assessment of Cancer Therapy, and each item is rated on a 5-point Likert scale [22]. The score ranges from 0, “not at all” to 4, “very much”, and the higher the score, the greater the severity of fatigue. The original FACT-F has been found to be reliable and valid [22], and the Korean version of the FACT-F subscale has been shown to be a reliable instrument to measure fatigue; the internal consistency reliability of FACT-F was found to be α = 0.91 in patients with cancer [23]. 

#### 2.4.4. European Organization for Research and Treatment of Cancer Quality of Life

##### Questionnaire Core 30

The Korean version of the European Organization for Research and Treatment of Cancer Quality of Life Questionnaire Core 30 (EORTC QLQ-C30) is a 30-item questionnaire used to assess the QoL of patients with cancer. There are 3 subscales consisting of global health status, functional scales (physical, role, emotional, cognitive, and social functioning), and symptom scales (fatigue, nausea and vomiting, pain, dyspnea, insomnia, appetite loss, constipation, diarrhea, and financial difficulties) [24,25]. All items scored from 1 to 4 points except for the 2 items in global health status that scored from 1 to 7 points [24,25]. The scores were converted to 100 points by scoring guidelines [26]. Reporting higher scores on the global health status and functional scale signifies higher QoL [25]. The internal constancy reliability of the Korean version of the EORTC QLQ-C30 was 0.80 for global health status, 0.87 for functional scale, and 0.84 for symptom scale [25]. 

### 2.5. Translation Process

The back-translation using Brislin’s model of translation [27] was used to translate the original CFS (English) into Korean. First, forward translation from English to Korean was conducted by two bilingual Korean native nurses working at medical centers in the United States. Second, a nurse who was not involved in the forward translation performed the back-translation of the Korean version into English. The research committee then reviewed both the original and Korean versions to ensure the meaning of the questionnaire items was correctly conveyed and verify the clarity and consistency of the language. Any issues identified were resolved through a consensus within the research team. Finally, the translated CFS was evaluated with five patients with cancer to assess the presence of grammatical errors, comprehensibility, and appropriate response time compared with the original version [12]. 

### 2.6. Statistical Analysis

All data were analyzed using the statistical program SPSS for Windows (version 23.0; IBM, Armonk, NY, USA). Descriptive statistics were used to describe general and medical characteristics as well as how patients rate cancer-related fatigue severity. Internal consistency reliability was evaluated using Cronbach’s α coefficient for all 15 items of the CFS. It was considered satisfactory if the Cronbach’s α coefficients were higher than 0.70. The Kaiser–Meyer–Olkin (KMO) test and Bartlett’s test of sphericity were conducted to test the goodness of fit of the data. An exploratory factor analysis (EFA) was performed with principal component methods based on eigenvalues, applying a varimax rotation with the 15 items of the CFS scale. A minimum factor load of r = 0.40 was considered for a particular factor, and the removal of items was considered when the absolute difference between the loadings was more than 0.20 [28]. Since item numbers 5, 8, 11, and 14 were reverse-coded for the original question, their reversed numbers were assigned. In addition, we performed a Spearman’s rank correlation analysis between the CFS-K subscales and total score and the assumed convergence criteria. 

The Spearman’s rank correlation coefficient was performed to assess the concurrent and construct validity between the variables measured using the CFS-K and those measured using the BFI-K, FACT-F, and EORTC QLQ-C30 relative subscales. 

## 3. Results

### 3.1. Reliability of Items

The reliability of all 15 items was analyzed; the Cronbach’s alpha coefficient for the CFS-K was found to be at an acceptable level (α = 0.806) in the patients with cancer. Upon omission of each item individually, the modified Cronbach’s alpha coefficients for all 15 items are described in Table 2. 

### 3.2. Exploratory Factor Analysis

The results indicated that KMO was 0.893 and Bartlett’s test of sphericity was significant (*p* < 0.001). The data were extracted into three factors (eigenvalue = 6.36; 1.80; 1.24) similar to the original fatigue scale. 

The three factors presented a delimited factorial distribution, each with at least three items. Eigenvalues > 1 were considered as a cutoff point of 0.40 for factorial loads both in relation to the proximity of the items in the analysis and adherence to the theory. 

The principal factor 1 (physical fatigue) was analyzed seven times and explained 42.43% of the variance, the second factor (cognitive fatigue) was analyzed four items and explains 11.99% of the variance, and the third factor (affective fatigue) was analyzed four items and explains 8.27% of the variance (Table 3). Two items (item 4 and 12) show no adjustments of the factor analysis compared with the original version of the CFS [12]. 

### 3.3. Reliability of the Subscales

The reliability analysis of the subscales based on the EFA showed that the Cronbach’s alpha coefficients were α = 0.908 for physical subscale, α = 0.830 for cognitive subscale, and α = 0.645 for affective fatigue. No corrected item-total correlation coefficient < 0.3 was found for the corrected item-total correlation, and so no item was deleted (Table 3). The correlations between subscales were shown to range from weak to strong (Table 4). 

### 3.4. Concurrent and Construct Validity

Table 5 shows the correlation matrix of the three subscales and the total score with other variables. The total and subscale scores of the CFS-K showed a significant correlation with cancer-related fatigue measured using the BFI (r = 0.27–0.73, *p* < 0.001) and fatigue and its impact on ADL measured by FACT-F (r = 0.36–0.80, *p* < 0.001). The FACT-F score had the highest correlation coefficient with CFS-K physical fatigue (r = 0.80, *p* < 0.001). 

For evaluation of construct validity, correlation coefficients were explored between the EORTC QLQ-C30 global score and CFS-K subscale and total scores, and physical, emotional, cognitive functioning and fatigue, pain, and appetite loss (Table 5). The CFS-K physical fatigue score revealed significant moderate to strong correlations with all other measures. On the other hand, the CFS-K affective fatigue and CFS-K cognitive fatigue scores displayed relatively weak correlations with all other variables.

## 4. Discussion

Given the high prevalence and multidimensional nature of fatigue in patients with cancer, there is a critical need for validated measures to assess and evaluate the impact of CRF. The objectives of this study were to translate the original English version of the CFS into Korean and examine the validity and reliability of the Korean version of the CFS in patients with various types of cancer.

For three dimensions of the Korean version of the CFS, the internal consistency of the physical, cognitive and affective subscales as well as that of the total scale was found to be acceptable. Compared to the original validation study and previous studies of psychometrics of CFS conducted using the Chinese, German, Greek, Brazilian, Persian and Turkish versions [13,14,15,16,17,19], the reliability of this study was similar except for the affective factor. The lower value of internal consistency for the affective factor in this study could be associated with the inherent complexity of the study sample, such as age, educational level, cancer type, and health condition. The inherent complexity of the participants may bring different comprehension and responses of the symptoms between individuals [17]; therefore, affective factors should be considered with the complexity of the participants. Since this study was performed in mixed groups of patients with cancer, it is likely to influence fatigue domains and associative factors and reduce sensitivity across patient groups. By this means, patients with different cancer groups and stages of cancer are unlikely to report the same level of fatigue as measured by severity, dimensions, and domains. Furthermore, although during translation, the choice of the best term was discussed and all steps of the guideline were strictly followed, differences between the original and translated version may be present because of cultural and linguistic discrepancies between the different countries. 

Difficulties were experienced in the forward–backward translation for items 4 (careless) and 12 (reluctant). Item 4, which relates to cognitive fatigue in the original version, was translated to imply performing a task with feelings of carelessness, so item 4 was classified as an aspect of physical fatigue. Item 12 (reluctant) was translated as not willing to do something or not wanting to make the effort to do something, and we interpreted this word to best align with cognitive fatigue in Korean rather than physical fatigue. These variations of factor loading also were found in previous studies conducted using the Chinese, German, Greek, and Brazilian versions of the CFS [13,14,15,17]. Similar to the present study, item 4 in the Chinese version also was interpreted as a physical, rather than cognitive, subscale. Item 11 had a higher loading on the cognitive fatigue subscale in the German version, and item 15 had high loading on affective fatigue. The Greek version of the CFS also showed variation in factor loading for item 9. In addition, according to the Brazilian version of the CFS, there was a strong correlation between the physical and cognitive subscales; therefore, item 4 was reduced because this item does not influence the cognitive subscale. These variations in the attributes of the original version indicate that fatigue is a complex and dynamic phenomenon that requires a differentiated view.

When evaluating the convergent validity of the CFS, there were moderate to strong positive correlations between CFS-K and BFI, FACT-F and EORTC fatigue. When related to the functioning capacities of EORTC QLQ-C30, there were moderate negative associations between CFS and EORTC QLQ-C30 functional scales. There is evidence to support the convergent/divergent validity of the CFS-K in patients with cancer in Korea. In addition, the results were compatible with that of the original validation study; higher CFS levels indicate lower quality of life. Furthermore, significant associations were identified between the CFS and EORTC fatigue, pain, dyspnea, insomnia and appetite loss. Convergent validity was demonstrated by the correlations between multiple measures, which are similar to previous studies. For instance, the convergent validity of the CFS was demonstrated with the EORTC QLQ-C30 in Greek and Turkish studies [15,16], with the Piper fatigue scale, Beck Depression Inventory, and Karnofsky performance scale in a Brazilian study [17], and with the Anxiety, Depression, and Karnofsky performance scales in a German study [13]. Based on the current findings, the CFS-K met the criteria for content, construct, and criterion validity as well as internal consistency. 

As for the utility, the ideal number of items remains unknown and may not be population-dependent or purpose-dependent. However, in general, multidimensional scales contain 30 items or more, which make completion challenging and a burden for those with severe fatigue [2]. Notwithstanding, the completion rate in the present study was adequate at 99.9%, which may be because this CFS-K questionnaire contains only 15 items. As fatigue is a multidimensional symptom, we believe that the CFS-K can evaluate fatigue from a multidimensional perspective in a more brief and practical manner compared with the other fatigue scales for use in patients with cancer. 

In conclusion, the findings of this study provide supporting evidence for the good internal consistency and construct validity of the Korean version of the CFS, as indicated by the results of convergent and divergent examinations. This aligns with a systematic review of CRF measurement questionnaires [2,11], which identified the CFS as one of the instruments with nearly ideal psychometric properties. However, it should be noted that the results of the exploratory factor analysis showed some differences between the original scales and the Korean version, which may be attributed to the complexity of the participants with various types of cancer. The diversity in clinical characteristics represents one of the limitations of this study. Additionally, since this study employed a cross-sectional design, it was unable to examine the longitudinal changes in CRF experienced by patients over time. Therefore, further research is warranted to test the Korean versions of the CFS in a more homogenous cancer population and to investigate the longitudinal stability and predictive validity of the scale. Furthermore, given the importance of aligning the definition and operationalization of fatigue in patients with cancer, qualitative approaches should be employed to explore the sub-domains of CRF that focus on the perceptions and perspectives of Korean patients with cancer. 

## 5. Conclusions

The results of this study demonstrate that the Korean version of CFS is a brief, valid, feasible, and practical instrument for assessing the multidimensional perspectives of CRF in patients with cancer. The findings highlight its potential value in enhancing our understanding of fatigue and evaluating the effectiveness of interventions aimed at managing fatigue in this population. However, considering the complexity of participants with various types of cancer, further testing of the Korean version of the CFS is warranted in more homogeneous cancer populations, including cancer survivors. 

## Figures and Tables

**Table 1:** Participant characteristics (*n* = 209).

Variable	Category	N (%)	Mean ± SD
Age, years			61.2 ± 11.6
Sex	Women	98 (46.9)	
	Men	111 (53.1)	
Marital status (*n* = 207)	Married	148 (71.5)	
	Single	17 (8.2)	
	Widowed	20 (9.7)	
	Divorced	22 (10.6)	
Type of cancer	Stomach cancer	65 (31.1)	
	Breast cancer	40 (19.1)	
	Colorectal cancer	34 (16.3)	
	Lung cancer	19 (9.1)	
	Esophageal cancer	10 (4.8)	
	Pancreatic cancer	5 (2.4)	
	Gynecological cancer	5 (2.4)	
	Prostate cancer	3 (1.4)	
	Oral cancer	3 (1.4)	
	Bladder cancer	4 (1.9)	
	Biliary cancer	3 (1.4)	
	Hematologic cancer	7 (3.4)	
	Double primary	6 (2.9)	
	Others *	5 (2.4)	
Stage (*n* = 197)	0	1 (0.5)	
	I	31 (14.8)	
	II	33 (15.8)	
	III	58 (27.8)	
	IV	74 (35.4)	
Therapies received at the point of questioning (*n* = 179) ^†^	Operation	52 (29.2)	
	Chemotherapy	155 (87.1)	
	Radiotherapy	31 (17.4)	
	Hormonal therapy	11 (6.2)	
	Immune therapy	6 (3.4)	

* Others included thyroid cancer (*n* = 2), liver cancer (*n* = 1), kidney cancer (*n* = 1), and cancer of unknown origin (*n* = 1). ^†^ Multiple responses allowed.

**Table 2:** Assessment of reliability using Cronbach’s alpha and Cronbach’s alpha if individual items were deleted (*n* = 209).

Item	Cronbach’s Alpha (α) If Item Deleted
	0.806
1 Easily tired	0.781
2 Having urge to lie down	0.777
3 Exhausted	0.773
4 Careless	0.774
5 Energetic feeling	0.830
6 Heavy and tired	0.778
7 Errors while speaking	0.779
8 Interest in something	0.828
9 Fed up	0.781
10 Forgetful	0.781
11 Ability to concentrate	0.834
12 Reluctant	0.782
13 Thinking has become slower	0.781
14 Encourage yourself to do something	0.830
15 Fatigue—you do not know what to do with yourself	0.786

**Table 3:** Modified model based on an exploratory factor analysis with subscales (*n* = 209).

Item Number and Content of K-CFS	Mean (SD)	α If Item Deleted	Factor Loading		
			Factor 1 (Physical Fatigue)	Factor 2 (Cognitive Fatigue)	Factor 3 (Affective Fatigue)
**Physical fatigue**					
3 Exhausted	2.73 (1.12)	0.882	0.882	0.159	−0.081
2 Having urge to lie down	2.95 (1.01)	0.893	0.808	0.201	−0.030
6 Heavy and tired	2.93 (0.98)	0.890	0.791	0.219	−0.137
1 Easily tired	3.08 (1.05)	0.894	0.782	0.156	−0.106
4 Careless	2.77 (1.10)	0.896	0.661	0.442	−0.106
9 Fed up	2.73 (1.09)	0.900	0.608	0.278	−0.346
15 Fatigue—you do not know what to do with yourself	2.55 (1.07)	0.901	0.607	0.364	−0.306
**Cognitive fatigue**					
13 Thinking has become slower	2.66 (1.10)	0.763	0.194	0.869	−0.031
10 Forgetful	2.72 (1.12)	0.767	0.242	0.804	−0.037
7 Errors while speaking	2.33 (1.12)	0.784	0.317	0.748	0.033
12 Reluctant	2.84 (1.02)	0.826	0.485	0.525	−0.263
**Affective fatigue**					
8 Interest in something	2.60 (1.01)	0.567	0.017	−0.179	0.714
5 Energetic feeling	2.93 (0.99)	0.574	−0.107	−0.007	0.689
14 Encourage yourself to do something	3.27 (1.00)	0.571	−0.120	−0.051	0.687
11 Ability to concentrate	3.11 (1.09)	0.591	−0.219	0.108	0.617
**Eigenvalue**			6.36	1.80	1.24
**Percentage of variance explained (%)**			42.43	11.99	8.27
**Cronbach’s alpha coefficient**			0.908	0.830	0.645

Abbreviation: K-CFS, Korean version of the Cancer Fatigue Scale.

**Table 4:** Inter-subscale correlation of Cancer Fatigue Scale factors in patients with cancer (*n* = 209).

	Physical Fatigue	Cognitive Fatigue	Affective Fatigue
r (*p*)			
Physical fatigue	-	-	-
Cognitive fatigue	0.66 (<0.001)	-	-
Affective fatigue	0.36 (<0.001)	0.23 (0.003)	-
Total score	0.93 (<0.001)	0.81 (<0.001)	0.57 (<0.001)

**Table 5:** Correlation * of the CFS-K with other variables (*n* = 209).

	CFS-K Physical Fatigue	CFS-K Cognitive Fatigue	CFS-K Affective Fatigue	CFS-K Total
BFI	0.76	0.55	0.27	0.73
FACT-F	0.80	0.52	0.36	0.76
EORTC QLQ-C30				
Global health/QoL	−0.53	−0.33	−0.37	−0.54
Physical functioning	−0.67	−0.48	−0.24	−0.64
Role functioning	−0.66	−0.47	−0.25	−0.63
Emotional functioning	−0.58	−0.42	−0.35	−0.60
Cognitive functioning	−0.52	−0.57	−0.31	−0.60
Social functioning	−0.42	−0.26	−0.19	−0.40
Fatigue	0.70	0.46	0.28	0.65
Nausea and vomiting	0.43	0.25	0.31	0.43
Pain	0.52	0.33	0.29	0.50
Dyspnea	0.52	0.30	0.28	0.50
Insomnia	0.46	0.28	0.22	0.43
Appetite loss	0.50	0.34	0.34	0.52
Constipation	0.35	0.18	0.23	0.34
Diarrhea	0.35	0.15	0.22	0.33
Financial difficulties	0.32	0.25	0.13	0.32

Abbreviation: BFI, Brief Fatigue Inventory; CFS-K, Cancer Fatigue Scale—Korean; EORTC QLQ-C30, European Organization for Research and Treatment of Cancer Quality of Life Questionnaire Core 30; FACT-F, Functional Assessment of Chronic Illness Therapy-Fatigue. * Correlation is significant at the 0.01 level for all values.

## Data Availability

The datasets generated during this study are available from the corresponding author upon reasonable request.
